# From PsO to PsA: the role of T_RM_ and Tregs in psoriatic disease, a systematic review of the literature

**DOI:** 10.3389/fmed.2024.1346757

**Published:** 2024-02-09

**Authors:** Bárbara Lobão, Diana Lourenço, Ana Giga, Pedro Mendes-Bastos

**Affiliations:** ^1^Instituto Português de Reumatologia, Lisboa, Portugal; ^2^Centro Hospitalar de Setúbal, Setúbal, Portugal; ^3^Janssen Portugal, Oeiras, Portugal; ^4^Dermatology Center, Hospital CUF Descobertas, Lisboa, Portugal

**Keywords:** psoriasis, psoriatic arthritis, disease interception, resident-memory T cells, regulatory T cells, IL-17, IL-23

## Abstract

**Introduction:**

Psoriasis (PsO) is a chronic skin condition driven by immune mediators like TNFα, INFγ, IL-17, and IL-23. Psoriatic arthritis (PsA) can develop in PsO patients. Although psoriatic lesions may apparently resolve with therapy, subclinical cutaneous inflammation may persist. The role of tissue-resident memory T-cells (T_RM_), and regulatory T cells (Tregs) that also contribute to chronic inflammation are being explored in this context. This systematic review explores T_RM_ and Tregs in psoriatic disease (PsD) and its progression.

**Methods:**

A systematic review, following Preferred Reporting Items for Systematic Reviews and Meta-Analyses (PRISMA) guidelines, was performed using Pubmed® and Web of Science™ databases on June 3^rd^ 2023, using patient/population, intervention, comparison, and outcomes (PICO) criteria limited to the English language.

**Results:**

A total of 62 reports were identified and included. In PsO, chronic inflammation is driven by cytokines including IL-17 and IL-23, and cellular mediators such as CD8^+^ and CD4^+^ T cells. T_RM_ contributes to local inflammation, while Tregs may be dysfunctional in psoriatic skin lesions. Secukinumab and guselkumab, which target IL-17A and the IL-23p19 subunit, respectively, have different effects on CD8^+^ T_RM_ and Tregs during PsO treatment. Inhibition of IL-23 may provide better long-term results due to its impact on the Treg to CD8^+^ T_RM_ ratio. IL-23 may contribute to inflammation persisting even after treatment. In PsA, subclinical enthesitis is perceived as an early occurence, and Th17 cells are involved in this pathogenic process. Recent EULAR guidelines highlight the importance of early diagnosis and treatment to intercept PsA. In PsA, CD8^+^ T_RM_ cells are present in synovial fluid and Tregs are reduced in peripheral blood. The progression from PsO to PsA is marked by a shift in immune profiles, with specific T-cells subsets playing key roles in perpetuating inflammation. Early intervention targeting T_RM_ cells may hold promising, but clinical studies are limited. Ongoing studies such as IVEPSA and PAMPA aim to improve our knowledge regarding PsA interception in high-risk PsO patients, emphasizing the need for further research in this area.

**Conclusion:**

Early intervention is crucial for PsO patients at high risk of PsA; T cells, particularly type 17 helper T cells, and CD8^+^ cells are key in the progression from PsO-to-PsA. Early targeting of T_RM_ in PsD shows promise but more research is needed.

## 1 Introduction

Psoriasis (PsO) is a chronic inflammatory skin condition characterized by thickened red plaques and silvery lamellar scales, primarily affecting the scalp, trunk and extensor surfaces of the limbs (1). The pathogenesis of PsO involves both environmental and genetic factors, with communication between various immune cell types via cytokines such as tumor necrosis factor alpha (TNFα), interferon gamma (IFN-γ), IL-17, IL-22, and IL-23, leading to the establishment of a self-sustaining inflammatory cycle (2, 3). Thus, PsO is a recurring immune-mediated skin disorder characterized by epidermal hyperplasia and the presence of extensive dermal inflammatory infiltrates, which are recognized as the key histologic features of psoriatic lesions (4). PsO is a T cell-mediated inflammatory disease and accumulating evidence suggests that T-helper (Th) cells play a pivotal role in its pathogenesis. Various subtypes of Th cells are involved in inflammatory responses (5). In psoriasis, intraepidermal T cells are predominantly CD8^+^ and represent key effector cells driving disease. These pathogenic cells produce IL-17 and the neutralization of CD8^+^T cells effectively prevents psoriasis development *in vivo* (6). Dermal CD4^+^ T cells produce inflammatory cytokines, such as IL-17A, IL-22, and IFN-γ, and are regarded as the main pathogenic T cell subpopulation (6).

One of the primary challenges encountered in PsO management lies in the reoccurrence of lesions at previously affected anatomical sites, either during inadequate treatment of the disease or after discontinuation of treatment after resolution of lesions. Existing evidence substantiates the idea of localized immune “memory” involving specific T cell populations that persists within cleared skin (7–11).

Psoriatic arthritis (PsA) develops in up to 30% of patients with PsO (12). Furthermore, over 90% PsA patients initially experience arthritis in the context of pre-existing PsO (13).

PsO condition runs a chronic course with recurrent disease flares and infrequent periods of prolonged drug-free remission (14). Immune inflammatory pathways associated with IL-23, IL-17 and TNF have been identified as also being relevant in PsA, which has led to the development of therapeutic agents targeting these cytokines (15). However, the great variability of response across different therapies suppressing specific inflammatory pathways is not yet fully understood (14, 15).

Strong evidence suggests that the initial phase of PsA is closely associated with early enthesitis, with subsequent inflammation spreading to involve the synovium (16). Studies have shown that many patients with PsO who have not yet experienced musculoskeletal complaints bear a significantly greater burden of subclinical articular inflammation when compared to healthy individuals (17, 18). In the progression from PsO to PsA there is a notable correlation with the development of synovitis and tenosynovitis, again tied to the synovio-enthesal complex (19). Growing evidence indicates that the pathogenesis of PsA may be rooted in autoimmune mechanisms. This is supported by significant links to class I human leukocyte antigen alleles, the development of ectopic lymphoid structures in synovial tissues containing T and B cell clusters, and the presence of clonally proliferating CD8^+^ T cells in both synovial tissue and fluid (20, 21). Nevertheless, the precise immunological mechanisms responsible for reducing immune tolerance and triggering the transition from PsO to PsA remain largely elusive (12, 22).

The recent discovery of a specialized subset of T cells known as tissue-resident memory T cells (T_RM_ cells) holds great potential for understanding the mechanism underlying of some chronic immune-mediated inflammatory diseases (9–11, 14). T_RM_ are a subset of memory T cells that play a crucial role in providing immune surveillance in specific tissues. They are identified by expression of CD69, a core signature marker of T_RM_ cells, and CD103 (2, 11, 23, 24). Under normal physiological conditions, T_RM_ cells reside in peripheral tissue sites (25).

Unlike other T cells, most T_RM_ cells permanently reside in tissues and do not circulate in the bloodstream. However, recent studies suggest that a small fraction of T_RM_ cells can exit tissues and persist in the blood, migrate to distant tissues or lymph nodes, or transform into other types of memory cell (26–28). T_RM_ can either differentiate from circulating precursors expressing the killer cell lectin like receptor G1 (KLRG1), or from central memory T cells (T_CM_) and effector memory T cells (T_EM_) in peripheral tissues. T_EM_ migrates to inflamed peripheral tissues, where they promptly display effector functions, whereas T_CM_ migrate to T cells regions within secondary lymphoid organs. There, they subsequently transform into T_EM_ and carry out effector functions. Within the local microenvironment, signals and cytokines originating from the tissue, such as transforming growing factor-beta 1 (TGFβ1), have the capacity to drive the tissue residency program in precursor cells (29–33). T_RM_ are primarily found in epithelial tissues such as the skin, lungs, and gut, where they functions to protect against infections and cancer. However, as previously mentioned, they may also play a role in disease through driving chronic inflammation (34, 35). T_RM_ have been implicated in heightened responses within inflamed environments due to their potential for crosstalk with other immune cells. The production of cytokines that recruit immune cells into inflamed tissues and the expression of genes involved in recruiting innate immune cells support the hypothesis that T_RM_ cells play a critical role in driving inflammation, contributing to the immune-inflammatory state (31, 36).

Human T_RM_ cells in the skin express the skin-homing chemokine receptors CCR4 and CCR10 as well as the tissue homing molecules CD103 and cutaneous lymphocyte antigen (CLA) (37). Skin’s T_RM_ cells actively search for pathogens by migrating within the epidermal compartment, utilizing several dynamic dendritic projections to squeeze between keratinocytes (38). After skin immunization, it has been suggested that the immune system may distribute antigen-reactive T cells both to the lymph nodes, as T_CM_ cells, and to the peripheral tissues, as T_RM_ cells (39).

While earlier studies primarily focused on CD8^+^ T_RM_ cells, it is essential to note that CD4^+^ T_RM_ cells also exist (40). CD4^+^ T cells assist in fostering the development of cytotoxic memory CD8^+^ T cells following infection (2).

A growing body of evidence suggests that T_RM_ cells can be found in the inflamed joints of patients with PsA (41). These cells are believed to play a crucial role in driving chronic inflammation and contributing to flares of this condition, similar to their role in driving recurrence of psoriatic skin lesions (14, 35, 42). However, T cells and their role in disease pathogenesis in entheses are poorly characterized. Nonetheless. healthy human entheseal CD4^+^ and CD8^+^ T cells express high levels of transcripts suggestive of tissue residency (41).

In addition to T_RM_ cells, regulatory T-cells (Tregs) represent another T-cell population that has gained attention in PsD. Tregs are a specialized immunosuppressive lineage of adaptative lymphocytes that have been shown to exert systemic effects in protecting tissues from. Tregs can suppress the activities of other immune cells mainly by secretion of IL-10 and TGFβ (43).

However, the literature regarding Tregs in patients with PsD is somewhat limited. Treg abnormalities such as decreased expression of CD39 and CD74, increased expression of IL-6Rα21, reduced suppressive functional capacity, and enhanced propensity to differentiate into cells that produce interleukin (IL)-17, have been observed in PsO patients. Only a few studies have investigated the role of Tregs in the pathogenesis of PsA and in-depth characterization of intra-articular Tregs is lacking (44). This systematic review aimed to assess the existing evidence regarding the role of T_RM_ and Tregs in PsO and PsA, and also in the progression from PsO to PsA.

## 2 Methods

This review adheres to the guidelines set by the Preferred Reporting Items for Systematic reviews and Meta-Analysis (PRISMA) (45). The PubMed® and the Web of Science™ databases were searched on June 3rd, 2023, using the strings “(psoriatic arthritis OR psoriasis OR nails OR enthesitis OR peripheral arthritis OR axial disease OR dactylitis OR scalp) AND tissue-resident memory T-cells”; psoriatic arthritis AND (regulatory T cells NOT tissue-resident memory T cells)”; “psoriasis AND psoriatic disease AND disease interception.” The search was restricted to papers written in English and involving human subjects. Results from these searches were combined and duplicate entries were removed. Two authors independently reviewed the abstracts for all publications identified, and in case of discrepancies, a third author made the final determination regarding the publication for this review. Reviews, case reports, and case series were excluded from consideration.

## 3 Results

**Figure 1:**
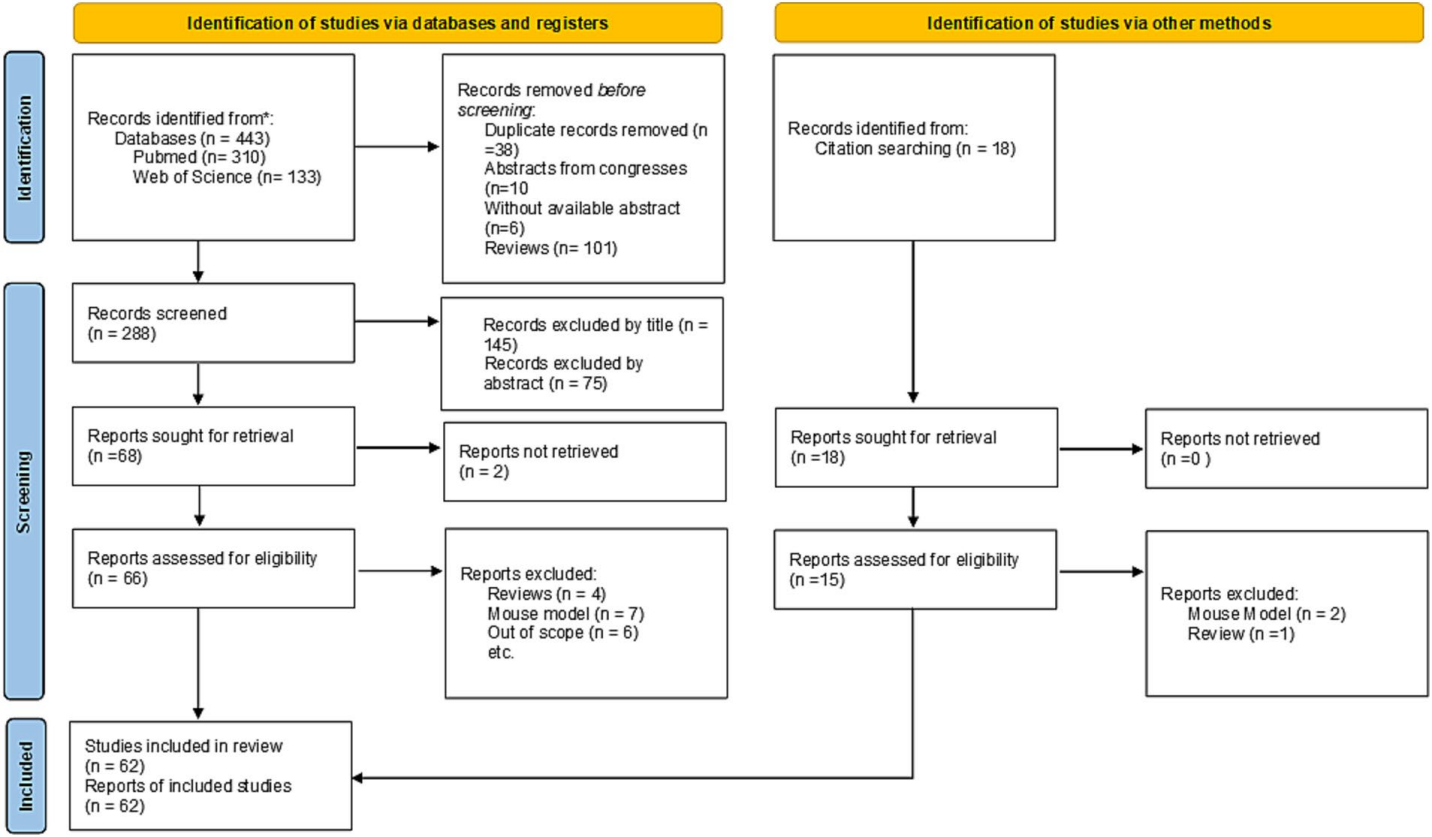
PRISMA flow for systematic review of the literature adapted from Page (45).

A comprehensive flow chart with the results of the literature search is illustrated in Figure 1. Initially, 443 records were exported from PubMed and Web of Sciences databases. Following elimination of duplicates and records lacking available abstracts, a total of 288 publications were retained. Of these, 220 were deemed ineligible for inclusion based on the application of selection criteria (145 by title, 75 by abstract review). Eighteen additional studies were identified through reference tracking, and citation. Ultimately, 62 studies were included in this review as summarized in Table 1.

**Table 1:** Summary of the studies included in the systematic review.

**Study**	**Methods**	**Main Results**
Wakita. H. 1994 (46)	5 PsO, without PsA or systemic disease Skin biopsy of central portion of the lesion and uninvolved skin (at least 5 cm apart from the involved skin)	Participation of memory T cells in the formation of the initial and active stages of PsO E-selectin and VCAM-1 on endothelium were critical adhesion molecules for initial trafficking of CD4^+^ memory T cells in psoriatic lesions
Homey, B. 2000 (47)	15 PsO 5 healthy individuals Skin biopsy: lesional and non lesional Peripheral blood sample	CCL20 and its receptor CCR6 are markedly upregulated in PsO CCL20 (induced in keratinocytes) and CCR6 may play a role in the recruitment of T cells to lesional psoriatic skin
Koreck, K. 2002 (48)	15 psoriatic patients 12 healthy donors Treatments applied: Dithronol, PUVA, Dithranol + PUVA, Dithranol + narrowband ultraviolet B (311 nm) and Re-PUVA Peripheral blood samples (before and after treatment for all patients)	No significant differences between total T cells, total B cells, Th cells, T cytotoxic cells and NK cells (before/after treatment and control) CD3^+^CD56^+^ NK cells of psoriatic patients were decreased compared to control: these cells could be actively involved in the development of Th1 mediated autoimmune diseases.
Curran, S. A. 2004 (49)	9 patients with PsA synovial tissue biopsies at the time of active disease CD4 and CD8 T cells characterization	76% of T cell clones in active tissue were polyclonal and unexpanded and decreased greatly with methotrexate 12% of the expanded clones could be grouped into clonal sets: exclusively CD8^+^ in lineage; persisted during methotrexate administration; were present in both joint fluid and blood- This implies that they were candidate driver clones that recognized an autoantigen The dominant feature of the disease was activation of multiple clones apparently lacking specificity for an inciting autoantigen
Vissers, W. H. P. M. 2004 (50)	8 PsO patients Biopsies from spreading psoriatic lesion from the distant uninvolved skin, the outer margin, the inner margin, and the central area	In the outer margin CD8^+^ and CD45RO^+^ T lymphocytes invade the epidermis From the outer to the inner margin, there was an increase of activation markers CD2 and CD25 and an increase of the cells expressing NK receptors (CD94 and CD161), and a ki67 increase Early phase of psoriatic process: CD8^+^, CD45RO^+^, CD2^+^ and CD25^+^ T cells Later phase of psoriatic process: CD94 and CD161 expressing cells together with epidermal proliferation
Clark, R. A 2006 (51)	Samples of normal human skin Isolation of T cells	T cells form normal skin had a diverse T cell repertoire, were largely Th1 biased Memory T cells are present in normal skin in substantial and previously unexpected numbers 98% of the CLA^+^ effector memory T cells are resident in normal skin and in resting conditions This could contribute to the development and perpetuation of inflammatory skin diseases
Piskin, G. 2006 (52)	Skin biopsy from PsO patients and normal individuals Isolation of keratinocytes	Keratinocytes produce IL-23 and this cytokine is sufficient to enhance IFN-γ production by type 1 memory T cells, perpetuating the inflammation process in the disease Low levels of keratinocyte-derived IL-23 were sufficient to enhance the IFN-γ production by memory T cells
Chen. Z. 2007 (53)	Healthy donors (PBMC) Naïve and memory CD4^+^ T cells were activated. Cytokine production was measured by ELISA and flow cytometry and mRNA was measured by qRT-PCT	In response to CD3/CD28 stimulation, memory T cells rapidly produced IL-17, naïve T cells expressed low levels TGF-β1 and IL-6 upregulated RORγt expression but did not induce Th17 differentiation of naïve CD4^+^ T cells. IL-23 promoted generation of human Th17 cells was a very potent inducer of other proinflammatory cytokines
Nograles, KE. 2008. (54)	16 PsO patients 5 healthy controls Skin biopsies and PBMC Analysis of T-cells: cytokine production by intracellular staining and flow cytometry; localization of the cytokine receptor in skin by immunohistochemistry and double-label immunofluorescence.	Th cells producing IL-17, IL-22 and/or IFN-γ are present in normal huma skin Keratinocytes stimulated with IL-17 expressed chemokines that are different from those induced by IFN-γ. IL-22 downregulated genes associated with keratinocyte differentiation and caused epidermal alterations
Pène, J. 2008 (55)	PsO tissue Characterization of infiltrated CD4^+^ T cells by cytokine production and gene profile analysis	Differentiated RORγ expressing T cells, producing high levels of IL-17, can represent up to 30% of infiltrating T lymphocytes. Activated Th17 cells produced IL-26, TNF-α, lymphotoxin-β, and IL-22. IL-17 and IL-22 concentrations secreted by tissue infiltrating Th17 cells could reach up to 100 nM and were inversely correlated with the production of Th1- and Th2-associated cytokines. Tissue-infiltrating Th17 cells was also characterized by high cell surface expression of CCR6 and by the production of CCL20/MIP3α Culture supernatants of activated Th17 cells, isolated from psoriatic lesions, induced the expression of gene products associated with inflammation and abnormal keratinocyte differentiation in an IL-17 and IL-22-dependent manner
Goodman, W. 2009 (56)	PsO patients (moderate disease) Healthy adult volunteers PBMC Purification of T lymphocytes: functional assays and IL-6R expression	IL-6 was necessary and sufficient to reverse T cell suspension by Treg IL-6Rα and gp130 expression was significantly elevated in psoriatic effector T cells compared to normal controls There were cells within lesional tissue that coexpress CD3, IL-17, and IL-6, indicating that Th17 cells are present *in vivo* within the psoriatic T_EM/EFF_ population and contribute to IL-6-mediated resistance to Treg suppression
Leipe, J. 2010 (57)	20 PsA patients with very early active PsA (2.3 months) healthy controls PBMC, cell culture after cells separation, total and naïve T cells Expression of T-lineage specific transcription factors and the response to CD4 T cells to Th17 cell inducing conditions	Frequency of Th17 cells and levels of IL-17 strongly correlated with systemic disease activity at both onset and the progression of PsA. Values were reduced to control levels in patients with treatment-controlled disease activity. Th17 cells were enriched in the joints, and increased frequencies of synovial Th17 cells expressed CCR4 and CCR6, indicative of selective migration of Th17 cells to the joints
Bovebnschen, H. J. 2011 (58)	PBMC from PsO patients and healthy controls CD4^+^ T cells were isolated based on flow-cytometry Tregs purified from CD4^+^ T Skin biopsies were analyzed by immunohistochemistry	Tregs of patients with severe psoriasis, compared to healthy controls, have an enhanced propensity to differentiate into IL-17A-producing cells on *ex vivo* stimulation Treg differentiation was linked to unexpectedly high RORγt levels and enhanced loss of Foxp3. IL-23 boosted Treg differentiation in psoriasis patients but less in controls IL-23 further reduced Foxp3 expression while leaving high RORγt levels unaffected
Kamiyama, T. 2012 (59)	PBMC from PsO patients and healthy donors CD4^+^ T cells were isolated based on flow-cytometry T_EM_ were analyzed by flow cytometry and ELISA	CCR6^+^CD146^+^ T_EM_ (CD4^+^CD45RA^−^CCR7^−^) cells had a greater capacity to produce IL-17 than CCR6^+^CD146^−^ T_EM or_ CCR6^−^CD146^+^ T_EM_ cells Co-expression of CCR6 and CD146 is a useful marker for Th17_EM_
Mommert, S. 2012 (60)	Memory T cells were polarized into Th17 cells H4R expression and function were analyzed by real time PCR, cytokine secretion assay of IL-17, and by electrophoretic mobility shift assay of activating protein-1	Th17 cells polarized by IL-1β together with IL-23 express the H4R on mRNA and protein levels IL-17^+^ were identified in positive lesions. IL-17^+^ lymphocytes were also positive for H4R. stimulation with histamine or a H4R agonist increased the production of IL-17 and induced activation protein-1 in Th17.
Tusda, K. 2012 (61)	5 PsO patients treated with ustekinumab on weeks 0,4 and 12 PBMC were isolated 1 month after the 3rd dose of ustekinumab 5 healthy controls	Ustekinumab improves clinical manifestations in patients with PsO without affecting production in memory T cells, T cell maturation, or T cell immune response repertoire
Yoo, I.S. 2012 (43)	47 PsO patients 47 healthy controls Peripheral blood CD4^+^ T-cells were isolated cultured and analyzed by flow cytometry and ELISA	There was no difference between patients and controls concerning the proportion of Th17 and Treg from PBMC PsO and PsA patients demonstrated higher proportion of induced Th17 cells, correlated with PASI score. IL-17A was higher in patients that in controls
Cheuk, S. 2014 (62)	Skin cell suspension from resolved psoriatic skin lesion from patients treated with narrowband-UVB, long-term TNF-α inhibition or IL-12/23 signaling inhibition Analyzed by flow cytometry, separated by cell sorting and T cells analyzed by quantitative RT-PCR	Epidermal T cells were highly activated in PsO and high proportion of CD8 T cells expressed T_RM_ markers In resolved PsO, a population of CLA, CCR6, CD103, and IL-23R expressing epidermal CD8 T cells were highly enriched Epidermal CD8 T cells expression CD103 responded to *ex vivo* stimulation with IL-17A production and epidermal CD4T responded in IL-22 production after as long as 6 years of TNF-α inhibition.
Skepner, J. 2014 (63)	Analysis of the RORγt inhibition in T cells isolated from PBMC and skin from psoriatic patients	TMP778 (selective RORγt inverse agonist) blocked human Th17 and CD8^+^ Tc17 cell differentiation and acutely modulated IL-17A production and inflammatory Th17 signature gene expression in mature human Th17 effector memory T cells. TMP778 inhibited IL-17A production in γδ T cells. IL-23 induced IL-17A production was also blocked by TMP778 treatment TMP778 selectively regulated Th17 signature gene expression in PBMC isolated from psoriatic patients
Fiocco, U. W 2015 (64)	SF from 16 patients with PsA to isolate SFMC PB from 7 healthy subjects to isolate PBMC CD4+ T lymphocytes were analyzed by flow cytometry IL-6 levels was measured by immunoassay	Expanded CD4^+^IL-17A-F^+^IL-23^+^Th17, CD4^+^CD25^−^Teff- and CD4^+^CD25^high^ Foxp3^+^ Treg subsets, showing similar levels of enhanced IL-6Rξ expression, were confined to PsA joints Complex interplay between IL-1, IL-6, and IL-23 signaling and differentiation of Th17 cells and CD4^+^ Tregs in sustained joint inflammation in PsA.
Sgambelluri, F. 2016 (65)	PBMC from PsO patients and healthy donors CRP quantification Phenotypically and transcriptionally characterization of T cells and	Circulation CD103^+^CCR4^+^CCR5^+^ and CCR4^+^CCR6^−^CD8^+^ T_eff_ cells were highly correlated with CRP cells and wells as PASI Contraction of circulation CCR5^+^ T cells in psoriatic patients, with highly significant CCR5^+^CD4 T cells and the PASI score Expression of *CCR5* and *CCL5* genes in psoriatic skin lesions was consistent with an accumulation of CCR5^+^ cells in psoriatic plaques
Soler, G. 2016 (66)	PBMC isolated from PsO patients and healthy donors analyzed by flow cytometry, oxidative stress measurements, ELISA, and immunohistochemistry	Mo-MDSC were elevated in psoriatic patients (in PBMC) Psoriatic Mo-MDSC directly suppressed CD8 T cell proliferation Psoriatic Mo-MDCS expressed reduced surface expression of PD-1 Psoriatic and control Mo-MDCS both induce Treg conversation from naïve T effector cells Treg induced by psoriatic Mo-MDSC displayed decreased suppressive functionality
Yang L. 2016 (67)	Psoriatic patients Healthy patients Expression and phosphorylation of STAT3 in psoriatic Tregs were evaluated by flow cytometry	Tregs from peripheral blood of psoriatic patients showed decreased suppressive function, along with phosphorylation of STAT3 Tregs from psoriatic patients can produced IFN-γ, TNF-α, and IL-17. IL-6, IL-21, and IL-23 induced the phosphorylation of STAT3 in Tregs
Cheuk, S. 2017 (34)	Skin biopsies from PsO and non-PsO patients *Ex-vivo* skin cell-cultures -Cell suspension analyzed by flow cytometry, sequencing, transcriptome analysis Cell sorting of tissue-derived cells - Cytotoxic assays	CD49a expression marks CD8^+^ T_RM_ cells poised for IFN-γ production in human skin IL-15 drives potent cytotoxic capacity in CD49a^+^ T_RM_ cells IL-17 is preferentially produced by CD49a^−^CD8^+^ T_RM_ cells in the skin CD49a^+^ versus CD49a^−^ T_RM_ cell functional dichotomy is preserved in PsO
Diani, M. 2017 (68)	PsO patients Phenotype of T cells, correlation with clinical parameters and bioinformatic analysis of gene expression data in psoriatic skin	CCR6^+^ CD4^+^ T_EM_ and T_EFF_ cells significantly correlated with systemic inflammation. The percentage of CXCR3^+^ CD4^+^ T_EM_ cells negatively correlated with the severity of the cutaneous disease CLA^+^ CD4^+^ T_CM_ cells expressing CCR6^+^ or CCR4^+^CXCR3^+^ negatively correlated with PsO severity Gene expression data showing marked increase of *CCR7* and CLA-encoding gene *SELPLG* expression in psoriatic skin and strong association of their expression.
Khairutdinov, V. R. 2017 (69)	41 patients with progressive PsO lesions 18 of these patients, also during the remission of the disease 16 healthy controls - Evaluation intradermal proliferation of T-cells and the number of CD45RO^+^ T cells in the skin	Progressive psoriatic lesion have Ki67 staining in keratinocytes and in the CD3ε^+^ cells of the dermal infiltrate Medium count of CD45RO+ cells per microscopic filed was 15 in healthy controls, 59 in patients in remission and 208 in patients with progression psoriatic patients
Wang, X. Y. 2017 (70)	189 PsO patients (95 severe disease, 94 moderate disease) 109 healthy individuals CD4^+^ T cells and Th17/Treg were extracted	Patients with severe disease had higher miR-200 expression, RORγt mRNA expression, percentage of Th17, Th17/Treg ratio and levels of IL-17 and IL-23 than moderate group Patients with severe disease had lower FOXP3 mRNA expression, and the percentage of Treg and TGF-β.
Baricza, E. 2018 (71)	PsA patients Healthy donors Study of Th17 cell differentiation	*RORC*, *TBX21*, *CCR6*, and *CCR4* expression of memory T cells were increased in healthy individuals compared to the naïve cells Cytokine-induced IL-17A production was different in PsA patients compared to healthy donors Naïve CD4 T lymphocytes are predisposed to differentiate into Th17 cells
Esmaeili, B. 2018 (72)	10 PsO patients 10 controls Isolation of memory T cells and analysis by flow cytometry. Determination of IL-23, IL-6, TNFα, TGFβ, and IL-17.	There was no significant difference in IL-17^+^ memory regulatory T cells between patients and controls Decreased percentage of IL-17 producing CD26^hi^ effector memory T cells in patients with no association with disease severity No differences in the cytokine levels
Sérézal G. I. 2018 (73)	Human skin from PsO lesions Healthy skin from PsO lesion Resolved PsO lesions Healthy skin from healthy donors Analyzed by immunohistochemistry, confocal imaging, flow cytometry, keratinocyte culture, and transcriptomic analysis	Resident T cells, isolated from the blood circulation, have the potential to create a proinflammatory environment in skin from PsO patients. T cells are a local source of cytokines in resolved PsO T cells could be responsible for local relapse by posing the whole tissue toward an IL-17A response
Bridgwood, C. (2019) (74)	15 enthesis from normal spinous process enthesis from patients who were undergoing spinal decompression or surgery from scoliosis correction of thoracic or lumbar vertebrate. Evaluated by immunohistochemistry, stimulated and analyzed for the production of disease relevant mediators	Human enthesis contains a myeloid cell population CD14^+^ population was the dominant entheseal producer of IL-23, IL-1β, TNF and CCL20
Diani, M. 2019 (75)	28 Pso patients 15 PsA patients 26 healthy subjects Analyzed phenotype and cytokine expression in circulating and synovial fluid T cells	Increased CD8^+^CCR6^+^ T cells effectors expressing CD69 and IL-17 producing T cells in patients with PsA CD8^+^ effector/effector memory T cells showed increased migration towards synovial fluid There was an accumulation of CXCR3^+^CD8^+^T cells and CD69^+^ cells in synovial fluid
Esmaeili, B. 2019 (76)	10 PsO patients 10 healthy controls CD4^+^ Memory T cells redox evaluation by flow cytometry, FRAP assay and real-time PCR.	Increased intracellular ROS production in memory CD4^+^ T cells of patients compared to controls Decrease catalase gene expression in patients These redox abnormalities have to relationship with IL-17 response in memory T cells.
Kampylafka, E. 2019 (77) (IVEPSA study)	20 PsO patients, without PsA, with nail or scalp involvement of PASI >6 as well as inflammatory or erosive changes in MRI or CT Patients were treated with SEC over 24 weeks	Skin disease, arthralgia, total PsAMRIS, and synovitis subscore significantly improved after 24 weeks Erosions and enthesophytes did not progress and bone mass in the distal radium significantly increased after 24 weeks
Klicznik, A. A. 2019 (26)	Skin explant cultures Analyzed by flow cytometry, sequencing	CD4^+^CD69^+^CD103^+^ T_RM_ in human skin can down-regulate CD69 and exit the tissue Skin tropic CD4^+^CD69^−^CD103^+^ population in human lymph and blood is transcriptionally, functionally, and clonally related to CD4^+^CD69^+^CD103^+^ T_RM_ population in the skin
Kurihara, K. 2019 (78)	10 PsO patients T cells expanded form *ex vivo* skin lesional biopsies Analyzed by flow cytometry and immnuofluorescence	Biopsed skin revealed CD8^+^CD20^+^ T_RM_ cells present in the epidermis of psoriatic lesions and acathosis. Sorted CD103^+^ T cells were mostly CD8^+^ memory T cells expression CD69 with a skin-homing potential. A part of CD8^+^CD103^+^ T cells produced interferon-γ, IL-17A or IL-22.
Vo, S. 2019 (79)	Non lesional sites of PsO Normal skin from non-PsO patients T_RM_ flow cytometry and immunofluoresence analysis	CD8^+^ T_RM_ cells with IL-17A-producing potential are accumulated in disease naïve non-lesional sites of PsO
Casciano, F. 2020 (80)	24 PsO patients 21 healthy subjects CCR4 expression in the different stages of memory T cells	The skin-homing CCR4 markers is mainly expressed in T_CM_ cells CCR4^+^ T_CM_ cells also express high level of CLA The more differentiated phenotype T_EM_ expresses CXCR3 and CCR5 but lower level of CCR4 and CLA Patients showed an expanded circulating population of CD8+ T_CM_ with phenotype CCR4^+^CXCR3^+^
Jesús-Gil, C. 2020 (81)	9 PsO patients 3 healthy controls Effect of IL-15 and IL-23 in CLA^+^ and CLA^−^ T cells from blood samples	There was a significant increase in IL-17F and IL-17A production in co-cultures of psoriasis skin-homing CLA^+^ T cells with epidermal cells when stimulated with IL-15 and IL-23 This response was reduced around 50 to 80% by blocking HLA class I and II molecules.
Loyal, L. 2020 (82)	PsO patients and healthy volunteers Blood and skin biopsies Analyzed by flow cytometry, mass cytometry, sequencing, RT-qPCR	Analogous CD8^+^ memory T-cell subsets exist: Tc2, Tc17, and Tc22 cells express IL-6R but not SLAMF7, completely lack cytotoxicity and instead display helper functions including CD40L expression CD8^+^ Th cells exhibit a unique TCR repertoire, express genes related to skin T_RM_ and are altered in the inflammatory skin disease PsO
Mashiko, S. 2020 (83)	50 subjects: 40 PsO patients with residual plaques treated with ADA or UST or SEC; 12 untreated patients and healthy controls Skin biopsies were characterized using mRNA expression, histology, and FACS of skin hematopoietic cells.	Analysis of treated PsO plaques revealed: Persistence of key PsO pathways such as granulocyte adhesion and diapedesis, Th17 activation pathway, and interferon signaling Focal decreases in parakeratosis and keratinocyte proliferation and differential reduction in IL-17 producing CD103^+^T cells No change in CD103^+^ T_RM_ T cells Mast cells were the dominant source of IL-22 in all psoriatic patients
Penkava, F. 2020 (21)	PsA patients Blood and synovial fluid samples were analyzed by flow cytometry, single-cell RNA-seq, TCE mapping, chemokine protein quantification	3-fold expansion of memory CD8^+^ T cells in the joints of PsA patients compared to PB. Pronounced CD8^+^ T cell clonal expansions within the joints expressing cycling, activation, tissue-homing, and tissue residency markers CXCR3 is upregulated in the expanded synovial CD8^+^T CXCL9 and CXCL10 (CXCR3 ligands) are elevated in PsA synovial fluid
Stell, K. J. A. 2020 (42)	PsA patients PBMC and SFMC were phenotypically, transcriptionally, and functionally analyzes by flow cytometry, sequencing, qRT-PCR, and Luminex or ELISA	IL-17A^+^CD8^+^ T cells were predominantly TCRαβ^+^ and their frequencies were increased in the SF versus PB of patients with established PsA TCRβ sequencing showed that these cells in the SF were polyclonal RNA-Seq and deep immunophenotyping revealed that PsA synovial Tc17 cells had hallmarks of Th17 cells (RORC/IL-23R/CCR6/CD161) and Tc1 cells (granzyme A/B). Synovial Tc17 cells showed a strong T_RM_ cell signature and secreted a range of proinflammatory cytokines. CXCR6 was identified as a marker for synovial Tc17 cells, and increased levels of CXCR6 ligand CXCL16 in PsA SF.
Wang, J. 2020 (84)	95 PsA patients from whom 22 received subcutaneous low-dose IL-2 peripheral Treg evaluation (CD4^+^ T cells)	PsA patients had lower peripheral Treg than healthy controls Treg numbers correlated significantly and negatively with the levels of disease indicators Low dose IL-2 combination therapy increased Treg numbers and relieved disease activity (DAS28 instrument)
Kasprowicz-Furmanczyk, M. 2021 (85)	32 PsO patients 10 healthy controls Immunohostochemistry of skin lesions and heathy skin	T_RM_ markers (CD4, CD8, CD103, CD69, CD49, CXCR6) and tissue expression cytokines (IL-17A, IL-22) were in higher amount in the epidermis and skin with psoriatic lesions compared with healthy skin There is a positive relationship between T_RM_ markers expression is psoriatic patients and the duration of skin lesions. - There is no relationship between the amount of T_RM_ and the severity of the disease.
Leijten, E. F. 2021 (86)	PsO, PsA patients, healthy controls In depth immunophenotyping of T cells and dendritic cell subsets Peripheral blood, synovial fluid, and skin biopsy were analyzed by flow cytometry, high-throughput transcriptome analysis and functional assays	Compared to healthy controls, peripheral blood of patients with PsA had: increased regulatory CD4^+^T cells; increased CD8^+^T cells IL-17A that produces IL-17A and IL-22 CD8^+^CCR10^+^ T cells were enriched in PsA and not in PsO CD8^+^CCR10^+^ T were endowed with a Tc2/22-like cytokine profile, lacked cytotoxic potential, and displayed overall regulatory function. Tissue-resident memory CD8^+^T cells derived from the skin are enhanced in the circulation of patients with PsA compared to patients with PsO alone.
Mehta, H. 2021 (87)	Same PsO lesions before SEC and GUS, non-lesional skin Flow cytometry analysis	Lesional versus non-lesional skin: Among CD11c^+^HLA^−^DR^+^ mononuclear phagocytes, CD64^bright^CD163^−^CD14^bright^CD1c^−^CD1a^−^ inflammatory monocyte–like cells were the predominant IL-23 producing cells together with CD64^−^CD163^−^CD14^−^IL-23p19^−^TNF-α inflammatory dendritic cell–like cells T cells – CD8^+^CD49a^+^ and/or CD103^+^ tissue-resident memory T cells, CD4^+^CD25^+^FoxP3^+^ regulatory T cells, and CD4^+^CD49a^−^CD103^−^ T cells were increased. Both treatments decreased the frequencies of inflammatory monocyte-like, inflammatory dendritic cell like, and CD4^+^CD49a^−^CD103^−^ T cells GUS reduced memory T cells while maintaining regulatory T cells SEC reduced regulatory T cells and maintain memory T cells
Mulder, M. L. M. 2021 (88)	45 PsO patients and 45 PsA patients Peripheral blood for flow cytometry analysis combined with machine learning	Key PsA- classifying cell subsets selected include (compared with PsO): increased proportions of differentiated CD4^+^CD196^+^CD183^−^CD194^+^ and CD4^+^CD196^−^CD183^−^CD194^+^ T cells reduced proportions of CD196^+^ and CD197^+^ monocytes, memory CD4^+^ and CD8^+^ T-cell subsets and CD4^+^ regulatory T cells Joint scores showed as association with memory CD8^+^CD45RA^−^CD197^−^ effector T cells and CD197^+^ monocytes
Nguyen, D.X. 2021 (89)	66 PsA patients (synovial fluid) 38 healthy volunteers (peripheral blood) Migration assay to study the inhibition of CCR6^+^ effector T-cell migration by Treg as a surrogate of Treg suppression of R cell migration	Treg enhanced effector T-cell migration toward CCL20 in psoriatic arthritis Treg from patients treated with anti-TNF suppressed CCL20-driven effector T-cell migration Increased Th17:Treg ration was reversed by anti-TNF in psoriatic arthritis
Phadungsaksawasdi, P. 2021 (90)	Skin biopsies from 9 psoriatic patients (active lesion, non-lesional skin, and resolved skin) at week 4 and 24 during SEC treatment Immunofluorescence and *ex-vivo* expansion of T cells	Active psoriatic epidermis contained PD-1 expressing CD8^+^CD103^+^ T cells possessed a canonical PsO-specific resident memory phenotype with IL-23R expression and produced IL-17A. Skin-infiltrating T cells were dominated by CD4^+^T cells, whiles CD8^+^ T cells, especially CD8^+^CD103^+^ T cells represented an oligoclonal population in active PsO PD-1 expression delineates a putative pathogenic subset of epidermal CD8^+^CD103^+^ T cells, which possibly play a role in PsO pathogenesis.
Raychaudhuri, S. K. 2021 (91)	10 PsA patients (PBMC and SFMC) Hi-D FACS to identify IL-17 in two subsets of T cells	Effector CD146^+^ (MCAM^+^) T cells were enriched at the synovial inflammation site in PsA CD146 was likely to be a key regulator for Th17 effector function in PsA This indicated a relationship between Th17 effector and CD4 and CD8 memory T cells and CD146 expression in inflammatory arthritis
Strobl, J. 2021 (28)	Patients undergoing allogenic peripheral blood HSCT Genotyping, mathematical modeling, single cell transcriptomics, and functional analysis of patient blood and skin T-cells	Detection of small consistent population of circulating skin-derived T cells with a T_RM_ in the blood Blood from patients with GVHD contained elevated numbers of host skin T_RM_ producing pro-inflammatory Th2/Th17 cytokines and mediating keratinocytes damage. Skin T_RM_ has the potential to reseed and propagate inflammation
Cook P. C. 2022 (92)	Skin biopsy of PsO lesions and healthy skin Single cell RNA-seq, and CITE-seq processing	Psoriatic Th17/Tc17 cells dampen an inflammation-suppressive program (*ZIST*) Knockout of *ZIST* transcripts ZFP36l2, ZFP36 in T cells indices Th17/Tc17 cytokines IL-23 blockade fails to normalize attenuated *ZIST* levels in healed PsO lesions Persistent *ZIST* suppression, even in therapeutically improved lesions, suggested it contributes to recurrence of disease after treatment
Kasprowicz-Furmanczyk, M. 2022 (93)	10 PsO patients and 10 healthy controls Immunohistochemistry of skin lesions and heathy skin after 12 weeks of therapy with calcipotriol and betamethasone dipropionate foam	T_RM_ markers (CD4, CD8, CD103, CD69, CD49, CXCR6) and tissue expression cytokines (IL-17A, IL-22) decreased in the epidermis on week 12. In the dermis only CD4 and IL-22 decreased. High expression of T_RM_ markers in the dermis may result in the rapid recurrence of the lesions after topical treatment discontinuation.
Liu, Y. 2022 (40)	8 skin samples from PsO patients Single cells profile CD45^+^ immune cell transcriptomes	Active proliferative expansion of the Treg and T_RM_ components and universal T cells exhaustion, with a relative attenuation of APC Skin resident memory T cells showed the greatest transcriptional dysregulation
Michalak-Stoma, A. 2022 (94)	52 male psoriatic patients 24 male healthy volunteers Multiplex cytokine array to measure various interleukins in serum	IL-6 and IL-9 concentration was high in patients than controls IL-23 concentration correlated negatively with the age of psoriatic patients and positively with psoriasis duration
Owczarczyk-Saczonek, A. 2022 (95)	13 psoriatic patients, 10 healthy skin samples Skin biopsies 3 time points (week 0, 4, and 12) after systemic therapy initiation with MTX, SEC, ADA T_RM_ markers evaluation	T_RM_ markers (CD4, CD8, CD103, CD69, CD49, CXCR6) decreased with the treatment, predominantly in the lesional dermis The most rapid response was observed with SEC at week 4 of treatment
Pouw, J. 2022 (44)	21 PsO patients, 21 PsA patients and 13 healthy controls Clinical data, sera, PBMC cells and SFMC (6 PsA patients)	Tregs from inflamed joints (compared to circulating counterparts): express increased levels of ICOS, CTLA-4 and TIGIT. have a distinct phenotype: IL-17A production and upregulation of CD161 and RORγt have a subset of with intermediated Foxp3 expression as the major cytokine producers and associated with an increased abundance of anti-ADAMSTSL5 autoantibodies. ICOS^+^ Treg associated with PASDAS (disease activity) Treg from inflammatory environment exhibit a distinct phenotype associated with loss of peripheral immune tolerance in psoriatic disease
Skougaard, M. 2022 (96)	70 PsA patients Frequencies of nine immune cell subtypes were determined by flow cytometry (PCA)	Four immune cellular phenotypes were discovered, exhibiting resemblance to 4 distinct components: explained 25.6% of the variance with contribution from Th17 cells, mTregs, dendritic cells and monocytes – was associated with longer disease duration and higher DAPSA driven by Th1, naïve Tregs and mTregs – was associated with shorter disease duration driven by both Th1, Th17 and CD8^+^ T cells characterized by a reverse correlation between CD8^+^ T cells and natural killer cells The complex immune cellular mechanisms in PsA allow the possibility of improving PsA patients’ stratification based on clinical and immune cellular phenotypes.
Ghaffarinia, A. 2023 (97)	7 psoriatic patients Skin biopsies: never-lesional and resolved skin punch biopsies	Reduced 5-mC and 5-hmC amounts (epigenetic markers) and decreased mRNA expression of TET3 enzyme in the resolved epidermis *SAMHD1*, *C10orf99*, and *AKR1B10* genes are highly dysregulated in resolved epidermis and the DRTP was enriched in WNT, TNF, and mTOR signaling pathways DRTP of keratinocytes may contribute to site-specific local relapse
Poloveri, G. 2023 (14)	8 patients with PsA and 5 patients with RA T cell isolation from synovial fluid mononuclear cells	Human arthritic joints contain distinct subsets of T_RM_ cells CD8^+^CD69^+^CD103+ T_RM_ cells: cytotoxic and Treg like T_RM_ cells are present in both PsA and RA Psoriatic and rheumatoid arthritis joints differ in T_RM_ subset composition Psoriatic arthritis is enriched for pro-inflammatory type 17 T_RM_ CD8^+^ cells with a pro-inflammatory cytokine profile (IL-17A^+^TNFα^+^ IFN-γ^+^) Rheumatoid arthritis is enriched for T cells with a cytotoxic profile

VCAM-2, Vascular cell adhesion molecule-1; CCL, C-C Motif Chemokine Ligand; CCR, C-C Motif Chemokine Receptor; NK, Natural Killer; Th1, T helper 1; CLA, Cutaneous lymphocyte antigen; IL, interleukin; IFN, interferon; T_RM_, resident memory T cells; Treg, Regulatory T cells; mTreg, memory Treg; PsA, Psoriatic arthritis; RA, rheumatoid arthritis; 5-mC, 5-methylcytosine; 5-hmC, 5-hydroxymethylcytosine; TET3, ten-eleven translocation; PCA, principal component analysis; Th17, T helper 17; DAPSA, Disease Activity in Psoriatic Arthritis Score; PBMC, peripheral blood mononuclear cells; SFMB, synovial fluid mononuclear cells; ICOS, inducible T cell costimulatory; CTLA-4, cytotoxic T lymphocyte-associated antigen 4; RORγt, retinoid orphan receptor gamma t; ADAMSTSL5, A Disintegrin and Metalloproteinase with Thrombospondin motifs -like protein 5; MTX, methotrexate, SEC, secukinumab, ADA, adalimumab; APC, antigen-presenting cells; Tc, cytotoxic T cell; ZIST program, transcriptional single-cell identity involving multiple inflammation-suppressive regulators; SEQ, sequencing; CITE, cellular indexing of transcriptomes and epitopes; HSCT, Hematopoietic stem cell transplantation; GVHD, Graft versus host disease; MCAM, melanoma cell adhesion molecule; GUS, guselkumab; SF, synovial fluid; PB, peripheral blood; CXCR, C-X-C Motif Chemokine Receptor; FACS, Fluorescence activated cell sorting; UST, ustekinumab; SLAMF, signaling lymphocytic activation molecule; T_CM_, central memory T cells; T_EM,_ effector memory T cells; PsAMRIS, Psoriatic Arthritis Magnetic Resonance Imaging Score; T_EFF_, effector T cells; Mo-MDSC, CD14^+^ HLA-DR^neg/low^ monocytic Myeloid-Derived Suppressor Cells; CRP- C-reactive protein; H4R, Histamine H4 receptor; MIP3α, Macrophage Inflammatory Protein-3; PUVA, psoralen and UVA.

### 3.1 Psoriasis

**Figure 2:**
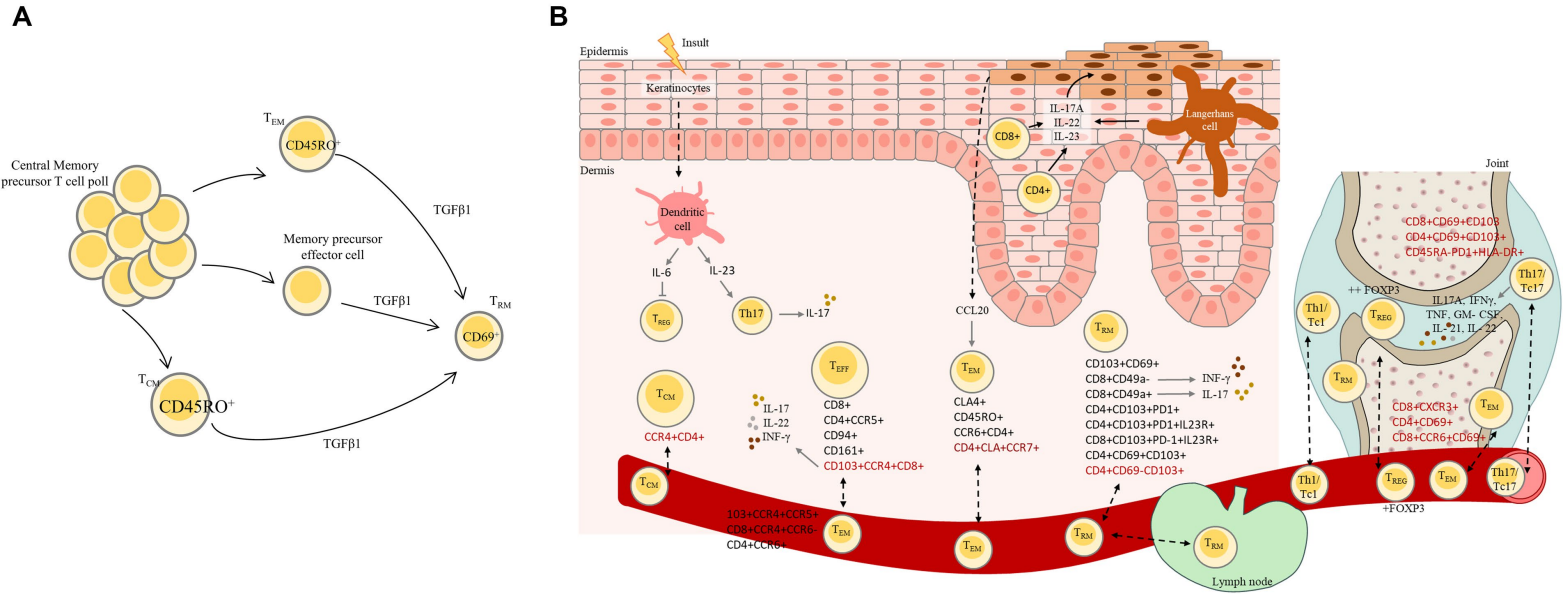
**(A)** Resident-tissue T cell differentiation from central memory precursor T cell pool. Central memory T cells are typically found in the bloodstream and lymphatic system. In psoriasis, these central memory T cells can undergo differentiation process and transform into resident tissue T cells (T_RM_) in the skin mainly through the action of TGFβ1. This differentiation process involves changes in the expression of surface markers such as CD45RO that is predominantly expressed on memory T cells and allow to distinguish from naïve T cells, chemokine receptors, and adhesion molecules, allowing central memory T cells to become specialized for residing in skin. **(B)** Psoriatic disease: initial physiopathology, disease recurrence in resolved psoriasis lesions and transition to PsA. Psoriatic disease involves a complex interplay of immune dysregulation, and environmental triggers. In PsO, activated T cells accumulate in the skin leading to inflammation and the release of cytokines, namely IFN-γ and IL-17, through IL-23 stimulation. These cytokines and immune response in PsO lead to the rapid proliferation of keratinocytes. Psoriasis can relapse in previously resolved lesions due to various factors, and some individuals with PsO may develop PsA as results of shared immune mechanism. Disease recurrence could occur due to the fact that the initial treatment does not resolve the underlying immune dysregulation. This is a complex phenomenon that involves interactions between various immune cells, including T_RM_ and Tregs. T_RM_ cells have been found to be present in the skin; however, they were also found in circulation, indicating that these cells can circulate between lymphoid and non-lymphoid tissues. In psoriatic disease Tregs are dysfunctional or reduced in number, leading to an imbalance between proinflammatory and anti-inflammatory responses that contribute to the recurrence of psoriatic lesions. In this process Langerhans cells are capable of producing inflammatory cytokines such as IL-17A that contribute to the perpetuation of inflammatory response and potentially lead to the recurrence of psoriatic lesions. In PsA, activated T cells produce TNF-α, IL-17, and IL-23. These cytokines play a central role in the inflammatory cascade, leading to joint inflammation, damage, and the clinical features of PsA. CD4^+^ and CD8^+^ T cells are recruited to the synovial tissue of affected joints in PsA patients. Specific T cells subsets capable of entering the bloodstream and recirculating between organs are represents in red in the figure. T_EM,_ Effector memory T cells; T_CM,_ Central memory T cells; Treg, Regulatory T cells, T_EFF,_ Effector T cells.

Chronic inflammation in PsO is characterized by tissue injury caused by activated immune cells, namely lymphocytes, which, through the production of a distinctive set of cytokines, contribute to disease pathogenesis (Figure 2) (55). The IL-23/IL-17 axis plays a central role in the immunopathogenesis of PsO by stimulating keratinocyte hyperproliferation and perpetuating T cell-mediated inflammation (55).

Do T_RM_ and Treg cells play any additional role in PsO pathophysiology?

The involvement of Effector Memory T (T_EM_) and Th cells in PsO has been described since 1994 (46). T_EM_ (CD62L^lo^/CCR7^lo^) are CD8^+^ cells that express CD45RO and that have greater cytolytic capacity than central memory T cells (T_CM_), and express integrins and chemokines necessary for localization to inflamed tissues (50). Th cells are CD4^+^ and play an important role in adaptive immunity (46). It has been shown that the initial phase of PsO is characterized by heavy epidermal infiltration of CD4^+^ T cells. Furthermore, these cells play a central role in the progression of PsO (43, 46). Helper CD8^+^ T cells may induce secretion of cytokines by neighboring cells, such as CCL20 from keratinocytes, which has been associated with the worsening of PsO (82). Additionally, helper CD8+ T are capable of strong expression of CD40L ligand upon activation, which could be a driver of autoinflammatory processes (82).

Treg cells are a subtype of Th cells that are characterized by Foxp3 expression, a transcription factor that is required for their development and function. These cells are crucial for suppressing immune responses mainly through secretion of IL-10 and TGFβ to maintain or restore immune balance. However, in a study by Yang et al., Tregs from psoriatic patients demonstrated a preponderance towards STAT3 phosphorylation when exposed to pro-inflammatory cytokines (such as IL-6, IL-21, and IL-23), resulting in a weakened capacity to suppress T cell mediated inflammation (67). Additionally, Tregs from patients with severe PsO have been shown to have an enhanced propensity to differentiate into IL-17A-producing cells, when compared to healthy controls, which may be triggered by IL-23. Furthermore, IL-23 may reduce Foxp3 expression, without impacting RORγt expression (58). The balance between Foxp3 and RORγt expression may determine whether Tregs maintain an immune-modulatory profile or undergo IL-23 driven differentiation into Th17 cells (i.e., a high RORγt:Foxp3 ratio will promote induction of proinflammatory IL-17A transcription program) (58). Th17 cells, which are IL-17 producing CD4 T cells, mediate inflammation and may also be involved in sustained inflammation later in disease progression (57).

CCL20 is believed to be a key mediator responsible for recruiting T_EM_ and Treg into PsO lesions (47, 55). The presence of CCL20 transcript, primarily expressed by keratinocytes, helps to distinguish lesional from non-lesional skin in psoriatic patients. Further, expression of CCL20 is higher in lesional skin when compared to non-lesional skin and can even be upregulated in non-lesional skin of psoriatic patients when compared to healthy skin from healthy controls. Moreover, CCL20-expressing keratinocytes colocalize with skin-infiltrating T lymphocytes and CCR6, the receptor for CCL20, is expressed at high levels on the skin homing cutaneous lymphocyte associated antigen (CLA^+^) subset of memory T cells (47, 55). CLA is a ligand for E-selectin and is typically present in T cells that infiltrate into cutaneous sites (51). In addition, CCL20 is associated with endothelial inflammation, which also reflects the state of systemic inflammation that characterizes PsO (47, 55).

IL − 15 has been proposed as a potentially relevant target against the IL-17 response in psoriasis, as it is co-expressed with IL-23 in psoriatic skin lesions. *IL-15* is also considered a susceptibility gene to the development of psoriasis. De Jesús-Gil *et al* identified a synergistic effect between IL-15 and IL-23 on IL-17A/F induced response in CLA^+^ T cells (81). Of note, this synergistic effect in IL-17A/F production was restricted to skin-related T_RM_ cells in psoriasis patients and it was not observed in healthy controls, and requires the presence of epidermal cells, indicating a cooperation between T cells and epidermal cells (81).

Involvement of specific T cell subsets may differ across different phases of PsO (50). Cytotoxic T cells (CD8^+^) and Effector T cells (T_EFF_ CD45RO^+^) infiltrate the epidermis during the early stages of development of PsO skin lesions accompanied by their increase in CD2 and CD25 activation markers (50). The heightened presence of CD8^+^ T cells in the epidermis at sites of initial formation of psoriatic plaques further supports the role of activated memory T cells in PsO lesions (46).

CCR5^+^ CD4 T cells also contribute to the formation of the PsO plaques (65). *CCR5* and *CCL5* genes expression in psoriatic skin lesions highlights the role of CCR5/CCL5 axis in PsO pathogenesis (65). CCR5 has been described as a mediator of CD4 T cell retention in dermal memory T cells clusters. CCL5 is produced by keratinocytes in psoriatic skin and play a role in recruitment of CCR5^+^ memory T cells (65). Furthermore, expression of CCR5 in psoriatic skin lesions is induced by proinflammatory cytokines such as IFN-γ, TNFα, and CCR5 serves as a chemoattractant for eosinophils, basophils, monocytes and T_EFF_ (CD4^+^ and CD45RO^+^), NK cells, and immature dendritic cells (65).

It is well established that severe PsO is associated with systemic inflammation leading to comorbidities such as cardiovascular disorders, metabolic syndrome, among others. Circulating CD103^+^CCR4^+^CCR5^+^ and CCR4^+^CCR6^−^ CD8^+^ effector T cells (T_EFF_) cells were highly correlated with C-reactive protein (CRP) as well as with PASI (Psoriasis Area and Severity Index) score in a study by Sgambelluri et al (65). Another study, by Diani et al., demonstrated that the circulating fraction of CCR6^+^ CD4^+^ T_EM_ and T_EFF_ cells also correlates with systemic inflammation in PsO (68). Furthermore, CLA expression on T_CM_ CCR6^+^ and CCR4^+^ cells inversely correlates with the PASI score (68). The authors emphasize this finding, hypothesizing that specific subsets of skin-tropic CLA^+^ CCR7^+^ memory CD4^+^ T cells could recirculate toward psoriatic skin in proportion to disease severity (68). Sgambelluri et al. (65) also hypothesized that in chronically inflamed tissue, a subset of differentiated resident cells (CD8 T cells CD103^+^CCR4^+^ CD8^+^ T_EFF_)_,_ is released in the circulation contributing to systemic inflammation (65). However, while the T_EM_ and T_EFF_ subsets of CCR4^+^ CD4 T cells are associated with PsO severity they do not correlate with systemic inflammation (65). CCR4^+^ CD4^+^ T cells, primarily T_CM_, constitute a subset of cells that efficiently re-enter the circulation from the skin and may relocate to the skin compartment upon antigen re-exposure or in response to inflammatory signals (65). This suggests that involvement of these cells occurs primarily in skin disease, specifically in the context of PsO pathogenesis, and may not have the same potential for impacting systemic inflammation (65).

T_EM_ and T_EFF_ from PsO lesions are chronically activated and poorly suppressed by Treg which, by definition, are immunosuppressive cells (40, 56). In lesional PsO skin, IL-6, a proinflammatory cytokine which signals through Stat3, is produced by keratinocytes, fibroblasts, endothelial cells, macrophages, and Th cells, allowing T_EM/_T_EFF_ cells to escape Treg-mediated suppression (56). The levels of this proinflammatory cytokine is markedly elevated and expressed by CD31 endothelial cells and expressed by CD11c^+^ dermal dendritic cells (DC) of PsO lesions (56). Furthermore, IL-6 was necessary and sufficient to reverse human T cell suppression by Treg in an *in vitro* model (56). Phosphorylation of Stat3 in T cells in response to IL-6 due to presence of IL-6Rα contributed to Th17 differentiation from naïve T cells (56). In lesional PsO skin there are cells that coexpress CD3, IL-17, and IL-6 indicating that Th17 cells are present within psoriatic T_EM_/T_EFF_ population contributing to IL-6 mediated resistance to Treg suppression (56). Thus, the proinflammatory effect of IL-6 and the resistance to Treg suppression may plays a key role in the pathogenesis of psoriasis. On the other hand the differentiation of Th17 cells, orchestrated by IL-6-indiced Stat3 phosphorylation, further contributes to the inflammatory milieu in psoriatic lesions.

A recent study showed that active psoriatic epidermis contained PD-1 expressing CD8^+^CD103^+^ T cells that correlates with disease severity and histopathology (90). Moreover, these cells have a memory resident phenotype, as they express IL-23R and produce IL-17A (90). Thus, the expression of PD-1 in epidermal psoriatic cells could delineate a putative pathogenic subset of epidermal CD8^+^CD103^+^ T cells, which possibly play a role in PsO pathogenesis (90).

T_EFF_ are considered circulating precursors of T_RM_ (65). Once T_EFF_ are recruited to the epidermis, they may up-regulate expression of retention signals such as CD103^+^ and CD69^+^ and reside there as T_RM_ (65). The presence of T cells expression T_RM_ markers (CD4, CD8, CD103, CD69, CD49, CXCR6) in the epidermis and skin of patients with PsO lesions increased when compared to healthy controls (85). Furthermore, there is a significant positive relationship between the expression of T_RM_ markers in plaque PsO and the duration of skin lesions (85). In human skin epithelia cytotoxic CD8^+^CD49a^−^ T_RM_ produced interferon-γ (IFN-γ), whereas CD8^+^CD49a^+^ T_RM_ produce IL-17, both promoting local inflammation in the skin (34). It was also demonstrated that the presence of IL-23 is important to activate T_RM_ to produce IFN-γ. Therefore, the augmented expression of IL-23 in keratinocytes and cutaneous APC may contribute to the perpetuation of the inflammation process in PsO (52, 53, 87). T_RM_ at barriers surfaces express the markers CD103 and/or CD69, which function to retain them in epithelial tissues (26). Characterization of skin T_RM_ within skin lesions showed that CD8^+^CD103^+^ T_RM_ cells, with a small number of CD4^+^CD103^+^ T_RM_ cells, were present in the epidermis of PsO and associated with acanthosis (78). These cells represent an effector population capable of maintaining the psoriatic phenotype and driving recurrence of disease at anatomical areas with cutaneous plaques (73, 90). Most of these cells express CD69, which conveys a skin-homing potential and CXCR3, contributing to the perpetuation of inflammation (68). T_RM_ cells are believed to be responsible for the immune memory of PsO skin lesions, may explain occurrence of skin lesions based on the Koebner phenomenon (85). A portion of CD8^+^CD103^+^ T_RM_ cells produced IFN-γ, IL-17A or IL-22 (78).

Contrary to the initial belief T_RM_ cells may not fully be characterized as resident in nature, as some may re-enter the recirculation from the skin to other tissues. CD4^+^CD69^+^CD103^+^ T_RM_ in human are capable of downregulating CD69 and exiting the skin (26). Furthermore, a CD4^+^CD69^−^CD103^+^ skin tropic population of cells was detected in lymph nodes and blood, and were transcriptionally, functionally, and clonally related to CD4^+^CD69^+^CD103^+^ T_RM_ in the skin. Additionally, Klicznik et al. demonstrated that CD4^+^ CD103^+^ T_RM_ could re-enter circulation and migrate to secondary body sites and reassume a T_RM_ phenotype (CD69^+^) (26). These findings raise the question of whether recirculating TRM cells derived from the skin may traffic to the musculoskeletal structures and contributes to the pathogenesis of PsA and the progression from PsO to PsA?

The complex relationship between dendritic cells (DC), keratinocytes, and T cells in PsO is highlighted by the dual production of IL-23, a central driver of inflammation. While primarily produced by DC, IL-23 can also be produced by keratinocytes. This cytokine plays a crucial role in enhancing IFN-γ production by memory T cells and promoting the expansion of pathogenic Th17 (52, 87). Notably, keratinocytes in lesional skin produced markedly higher levels of IL-23 compared to keratinocytes in normal and psoriatic non-lesional skin (52, 79).

In the ECLIPSE study, a head-to-head clinical trial comparing IL-23p19 blockade with guselkumab and IL-17A blockade with secukinumab for the treatment of moderate-to-severe psoriasis in adults (87). Although both guselkumab and secukinumab decreased the frequency of CD4^+^CD49a^−^CD103^−^ T cells, they exerted a differential effect on CD8^+^ T_RM_ and Tregs. By week 4, secukinumab reduced the frequency of Tregs but not CD8^+^ T_RM.,_ whereas guselkumab decreased the frequency of T_RM_ but not of Tregs (87). Therefore, an increase in the ratio of Treg to CD8^+^ T_RM_ was observed in lesional skin of patients treated with guselkumab compared to patients treated with secukinumab and this differences reached significal significance at week 24 (87). Although skin biopsies were not obtained at later timepoint in the ECLIPSE study, these finds could potentially account for guselkumab achieving superiority versus secukinumab in achieving the primary endpoint of PASI 90 response at week 48 (87).

### 3.2 Psoriatic arthritis

PsA is an inflammatory, MHC class I allele associated disease, in which injury is likely mediated predominantly by T cells (Figure 2) (42, 49). The enthesis is considered the cardinal lesion in PsA, and recently, Bridgwood C et al. showed that normal spinal enthesis soft tissue has resident myeloid cells capable of being induced to produce IL-23 (74). Naïve CD45RO- CD4+ T lymphocytes are predisposed to differentiate into Th17 cells, characterized by IL-17A and IL-22 production (71). In the very early stages of the disease, which is strongly associated with early enthesitis, IL-17 producing CD4^+^ Th17 cells, initiate inflammation and may also be involved in sustained inflammation later in disease progression (57). The unregulated of Th17 cell activity is due to intrinsically elevated expression of *RORC* gene (RAR Related Orphan Receptor C) accompanied by biased Th17 cell development and resistance of Th17 cells to natural antagonists, such as IL-12, IL-4 and IFN-γ (57).

To characterize the network of immune responses and chemokine signaling in the synovial microenvironment that could support the involvement of TRM cell in the pathogenesis of PsA Steel et al. conducted a comprehensive analysis of T cells derived from both peripheral blood and synovial fluid of patients with established PsA. The results showed that IL-17A^+^CD8^+^ T cells were predominantly TCRαβ^+^. Moreover, they were more frequent in the synovial fluid compared to peripheral blood (42). These cells were also polyclonal, and Tc17 cells (IL-17A^+^CD8^+^) express hallmarks of both Th17 cells (*RORC/IL-23R/CCR6/CD161*) and Tc1 cells (granzyme A/B). Synovial Tc17 cells exhibited a strong T_RM_ cells signature (CD45RA^−^PD1^+^HLA^−^DR^+^) secreting a range of proinflammatory cytokines (IFN-γ, TNF, GM- CSF, IL-21, and IL-22) alongside with IL-17A (42). Retention of these T_RM_ cells in the joints could be mediated by increased levels of CXCR6 ligand CXCL16 (42). CXCL16 possesses chemotactic and angiogenic properties and can be produced as a soluble mediator or as a transmembrane-bound chemokine by monocytes, macrophages, and dendritic cells. Furthermore, CXCL16 was shown to enhance recruitment CXCR6 expressing T cells derived from inflamed tissue (42). Diani et al. demonstrated that the proportion of CXCR6^+^CD8^+^T cell effectors expressing CD69, which are capable of trafficking to synovial fluid, was increased in the circulation of PsA patients and correlated with serum CRP levels (75). Furthermore, the number of IL-17 producing cells was also increased in the circulation of PsA patients (75). Moreover, accumulation of CXCR3^+^ CD8^+^ T cells, CD4^+^ T cells and CD69^+^ cells was found in synovial fluid from PsA patients (21, 75). Taken together, these findings suggest a potential role for CD8^+^ memory T cell effectors in both systemic and joint manifestations of PsA (75).

According to a study by Povoleri et al. T_RM_ cells may also play a fundamental role in PsA. CD8^+^CD69^+^CD103^+^ T_RM_ cells, such as cytotoxic Treg like T_RM,_ and CD161^+^CCR6^+^ type 17-like T_RM_ cells with a pro-inflammatory cytokine profile (IL-17A^+^TNFα^+^ IFN-γ^+^) were identified in the synovial fluid of PsA patients. Only one population of CD4^+^CD69^+^CD103^+^ T_RM_ cells was detected in synovial fluid of PsA patients (14). The significantly larger fraction of IL-17A-secreting tissue-resident and non-resident CD8^+^ T cells within synovial fluid PsA may contribute to the persistence of the disease (14).

Tregs, particularly those expressing an immunosuppressive profile (i.e., CD4^+^CD45^+^ Foxp3^+^) have also been implicated in the pathogenesis of PsA (84). In a study by Wang et al. PsA patients had lower peripheral Treg counts compared to healthy controls, which was accompanied by an increase in Th17 cells. Also, the absolute number of peripheral Treg cells was significantly negatively correlated with PsA disease activity (84). A very recent study demonstrated that Treg cells derived from the inflammatory microenvironment of inflamed joints in patients with PsA are phenotypically and functionally different from Tregs in the circulation (44). Tregs can down regulate their key transcriptional factor Foxp3 and to assume T_EFF_ phenotype and functions accompanied by the production of pro-inflammatory cytokines including IL-17, upregulation of inhibitory immune receptors, and upregulation of CD161, RORγt and ICOS (44). Treg cells that downregulate Foxp3 exhibit lower expression of inhibitory receptors such as CTLA-2 and TIGIT and produce even more IL-17, displaying the highest expression of CD161 (44). Furthermore, a transcriptional network profile identified through *ex vivo* signaling protein mapping on T lymphocytes in PsA joints revealed the complex interplay between IL-1, IL-6, and IL-23 signaling and differentiation of Th17 cells and CD4^+^ Tregs in sustained joint inflammation in PsA (64, 71).

### 3.3 PsO-PsA progression

The transition from PsO to PsA has gained interest in recent years. However few studies address the roles of T lymphocytes, namely CD4^+^ and CD8^+^ cells after PsO onset and before clinical manifestation of PsA. CD4^+^ T_EM_ cells expressing Th17-associated trafficking receptor CCR6 have been positively correlated with systemic inflammation in patients with psoriatic disease (PsO or PsA) (75).

An analysis of chemokine receptor profiling of circulating cells revealed a reduction in the percentage of CD8^+^ and CD4^+^ T cells expressing the Th1/Tc1-associated trafficking receptor CXCR3^+^, in patients with compared with patients with PsO limited to the and healthy subjects (75).

Another study by Leijten et al. showed that the peripheral blood of patients with PsA, when compared to healthy donors, was characterized by an increase in regulatory CD4^+^T cells and IL-17A and IL-22 coproducing CD8^+^ T cells (86). Further, CD8^+^CCR10^+^ T cells were enriched in the peripheral blood of PsA patients compared to PsO patients (86). These cells are specific T_EM_, with a Tc2/22-like cytokine profile (CD8^+^T cells) and regulatory function. They also co-express skin-homing receptors CCR4 and CLA, along with high levels of DNAX accessory molecule 1. Additionally, these cells were detected under both inflammatory and homeostatic conditions in skin, but were not enriched in synovial fluid (86). Furthermore, this CD8^+^ T cell subset exhibit a transcriptomic profile comparable to that observed in T_RM_ cells, that originated in the skin, but not in the joint (86).

PsA patients also exhibited a decline in the percentage of circulating Th1/Tc1 IFN-γ^−^ producing CD8^+^ and CD4^+^ T cells compared to patients with PsO (75). Findings from Diani *et al* supported the concept of CXCR3^+^ cells recruitment to inflamed psoriatic tissue. Based on analysis of the T cell characteristics present in both peripheral blood and synovial fluid mononuclear cells, which demonstrated accumulation of CXCR3^+^ T cells. Notably, a substantial increase in the levels of CXCR3 ligand, CXCL10, was identified within synovial fluid (75).

The use of machine learning for analysis of peripheral blood immune cell phenotype of consecutive PsO and PsA patients allowed discrimination of immune profiles (88). Key PsA-classifying cell subsets identified included increased proportions of differentiated CD4^+^CD196^+^CD183^−^CD194^+^ and CD4^+^CD196^−^CD183^−^CD194^+^ T-cells and reduced proportions of CD196^+^ and CD197^+^ monocytes, memory CD4^+^ and CD8^+^ T-cell subsets and CD4^+^ Treg cells (88). The reductions in these T cell subsets could be explained by migration of these cells to the joints and entheses of PsA patients, possibly contributing to disease progression from PsO to PsA.

## 4 Discussion

Over the past decade, the critical involvement of T cells in the development of PsO skin lesions has been extensively elucidated. Our understanding of the distinct contributions of various T cell subsets to the pathogenesis of PsO has significantly advanced. In particularly, T cells producing IL-17A, play a central role in driving PsO (34, 42, 62, 63, 68, 75, 76) and mature CD8^+^ T cells could be a putative link to systemic inflammation (75).

Activated T cells can migrate into the epidermis and recognize epidermal autoantigens and possibly progress toward differentiating into T_RM_ (7). That T_RM_ are found in the systemic circulation suggests that re-activated T_RM_ are capable of retrograde migration from non-lymphoid tissue, such as the skin. In turn, these circulating T_RM_ retain the capacity to return to the skin. Furthermore, these cells were also identified in patients with chronic inflammatory diseases, such as psoriatic disease, leading to the hypothesis that T_RM_ regress from inflamed tissue as well (36). The presence of T_RM_ in both tissue and circulating compartment could have implications for development of novel therapies targeting chronic inflammation and circulating T_RM_ could provide a vital diagnostic tool as biomarkers of disease activity and even potentially predict resistance to therapy (36). Additionally, complete T_RM_ suppression appears to be required for achieving disease remission (85).

Towards this, there is a paucity of research assessing the dynamic changes in T_RM_ counts, pathogenic versus non-pathogenic function induced by available psoriasis therapies (95). Compared to TNF inhibition and methotrexate, IL-17A inhibition achieves the most rapid reduction of expression of T_RM_ specific markers. Interestingly, after psoriatic lesions have resolved T_RM_ can still produce IL-17A and the implications for treatment to prevent relapses are unknown (98). It seems that it may vary depending on the therapy. Furthermore, the reduction of the expression of T_RM-_specific markers was observed predominantly in the dermis, and not in lesional epidermis (95). Thus, how topical drugs may influence T_RM_ quantity in psoriatic lesions remains a relevant question?

Mehta et al. in a study subanalysis of lesional biopsy specimens taken from patients successfully treated with either an IL-23p19 inhibitor, guselkumab, or an IL-17A inhibitor, secukinumab, observed that both CD4^+^ and CD8^+^ T_RM_ cells decreased in psoriatic lesions from baseline through week 24 in both groups; however, the decrease in CD8^+^ T_RM_ cells' frequency was significant only for patients treated with guselkumab (87). IL-23 receptor is reported to be upregulated in the CD8^+^CD49a^−^CD103^+^T_RM_ subsets relative to CD8^+^CD49a^+^CD103^+^T_RM_ (34). Further, CD49a^−^ T_RM_ cells preferentially produce IL-17 and these cells are profoundly expanded in the hypertrophic epidermis of psoriatic lesions (34). Which may, in part, account for the impact of the treatment with guselkumab.

T_RM_ has an undeniable ability to accumulate in the skin of psoriatic patients with significant longevity. Therefore, the relationship between T_RM_ accumulation and disease duration is also an important consideration. This could potentially explain the suboptimal therapeutic response in patients with longer disease duration, often accompanied byrapid recurrence of psoriatic lesions at the same anatomical sites (85).

Taken together, these considerations could support early intervention therapeutic in PsO specifically targeting T_RM_ cells to achieve better responses and remission of disease. Eyerich et al. hypothesized that patients with a short disease duration (≤2 years) may exhibit a more rapid and pronounced response to guselkumab treatment (99). Furthermore, these patients may be better positioned to maintain long-term drug-free disease control after guselkumab withdrawal (99). However, the data are sparce and disease modifying trials are warranted (7, 100).

Our extensive understanding of PsA primarily derives from studies conducted on well-established cases, focusing on patients who experienced having the disease for several years. However, our current knowledge of early-stage PsA remains limited, as early intervention represents an under-recognized clinical unmet need in PsA (77). Nevertheless, the most recent EULAR guidelines, which address the transition from PsO to PsA have formulated guidance in the areas of interception of PsA and the clinical management of patients at high risk development of PsA (101). Three distinct stages relevant to PsA considered: patients with PsO at higher risk of developing PsA, those with subclinical PsA and those with clinical manifestations of PsA (101).

In the absence of specific PsA biomarkers, early detection of PsA in PsO patients is both challenging and crucial for timely treatment and inhibiting structural damage (16, 102). Defining a specific immune profile and imaging modalities that could identify patients before onset of clinical features of PsA could potentially facilitate the referral to the rheumatology clinic (16, 85, 88, 93, 101). Moreover, early treatment of PsA may lead to better outcomes for patients due to enhanced therapeutic effectiveness (16, 101). The severity of skin involvement in PsA patients varies. Many but not all PsA patients may have PsO involving the skin requiring systemic treatment (16). Therefore, there is significant interest in the concept of treating the PsO to prevent PsA in a “Treat to Intercept” approach (16). A recent retrospective study published by Singla et al., which included a large cohort of patients with PsO demonstrated that treatment of PsO patients with IL-12/23 or IL-23 inhibitors was associated with reduced risk of inflammatory arthritis when compared with TNF inhibitors. No significant differences were found between IL-17 inhibitors and TNF inhibitors (103). Further studies are needed to confirm these results.

The convergent immunological bridge between the skin and joints is being increasingly recognized (16). Not activated T cells, including CD4^+^ and CD8^+^ T_RM_, are present at under normal conditions in both sites (14, 68, 75, 85, 86, 88, 90, 93, 95).

The increased frequency of IL-17A^+^CD8^+^T cells in synovial fluid of patients with PsA adds growing evidence that CD8^+^ T cells are relevant to the immunopathogenesis of PsA (42). A study by Poloveri et al. showed that CD8^+^ Treg-like cells harbors various markers of T_RM_ cells, suggesting that these cells may persist in the synovium joints in patients with PsA (14). Moreover, three clusters of T_RM_ cells were found in the psoriatic joints. Joints of PsA patients are enriched for pro-inflammatory type 17: CD8^+^CD69^+^CD103^+^ T_RM,_ characterized by the expression of CD161 and CCR6. The presence of these clusters of cells, all containing IL-17 producing cells suggests that they represent different fates or states of type 17-like T_RM,_ cells. This supports the concept that Tc17/T_RM_ cells in PsA are polyfunctional and able to produce multiple cytokines that drive PsA inflammation (14, 42). In turn, synovial type 17 T_RM_ cells may contribute to joint flares in PsA after treatment withdrawal as in PsO.

These findings lend strength to the idea that there are human CD8^+^ T_RM_ in circulation (26, 104), suggesting a potential role of these cells in the progression from PsO-PsA (42). This raises the hypothesize that PsO, as a human disease of the barrier tissue may involve a dynamic balance between tissue resident and recirculating memory T cells (105). CD4^+^ T cells also recirculating between blood and skin and could play a key role in determining the amplitude of skin inflammation in more severe PsO (68).

Some studies that included PsA patients evaluated CD8^+^ T and CD4^+^ T cells in the blood, synovial tissue, and synovial fluid showed that there are significant differences between these compartments (42, 75, 86). This is further supported by a study indicating that the subset composition and phenotype of peripheral blood T cells do not mirror that of synovial fluid, suggesting that blood represents a distinct compartment of T cells (23). Additionally, distinct trafficking patterns exist for T_RM_ subsets (23). The molecular profiles of resident and circulating T cells could offer insights into the relationship between the skin and joints (23).

The expression of CXCR6, a marker of T_RM_, could enhance residence of synovial Tc17 cells in inflamed tissue. This is accompanied by an increase in the levels of CXCL16 in PsA synovium (42). CXCR6 expression at the site of inflammation has been reported in PsO, and CXCL16 has been shown to enhance recruitment of inflamed tissue-derived CXCR 6 expressing T cells (42, 85, 106). A recent study has shown that CXCR6 is the most up regulated chemokine receptor in cutaneous lesions of PsA patients, and its expression correlates positively with PASI score (107). Moreover, CXCL16 blockade or CXCR6 deficiency led to reduced features of arthritis and lower IL-17 production in experimental models (108).

Thus, taken together, the data suggest that increased CXCR6 expression in PsO skin lesions of PsA patients may contribute to the recruitment and persistence of Tc17 cells in their inflamed joints (42, 85, 107).

Few clinical studies have addressed disease interception for PsA through early interventions in high risk PsO patients. The IVEPSA study is an open-label, prospective, exploratory interception study in very early PsA patients with subclinical inflammatory lesions in the joints, treated with an IL17A inhibitor (77). Data from this study demonstrated that inhibition of IL-17 halts the progression of catabolic and anabolic changes in patients' joints (77). Therefore, IVEPSA provides a strong rational for early interventions to intercept PsA. However, selection of high risk patients for early intervention could pose challenges (77). Identification of patients through the use of flow cytometry and machine learning algorithms, while not currently applicable in clinical practice, could offer new perspectives for accurately selecting patients based on their immune profile (88). Similarly, the PAMPA study is an ongoing multicentric, randomized, double-blind, placebo-controlled, interventional, PSA prevention trial at high risk for progression to PsA who are receiving treatment with guselkumab and non-biological standard of care (109). Taken together these studies will contribute to approaches for identifying patients at high risk of progression to PsA and the impact of treatment in preventing this process (109). Due to the limited number of available studies for this systematic literature review, particularly those addressing the transition PsO to PsA, the data on this topic are limited. While there are strong indications regarding the role of T_RM_ and Treg in intercepting in PsA and the potential for targeting them for intercepting PsA, clinical studies are needed to further explore and translate these described mechanisms to patients and potential treatment targets.

## 5 Conclusion

In conclusion, early intervention in PsA represents an under-recognized clinical need particularly in high-risk PsO patients. Early detection, although challenging without specific biomarkers, is crucial for timely treatment and to prevent permanent structural damage. Research has shed light on the role of T cells, particularly those producing IL-17A, in driving PsO and in the progression PsO to PsA. CD8^+^ cells and T_RM_ have emerged as significant players in both skin and joint inflammation. Targeting T_RM_ especially in the early stages of disease, appears promising for improving treatment outcomes and maintaining long-term disease control. While these findings offer promising avenues for early intervention and understanding the role T_RM_ and Tregs cells in PsO and PsA, the limited number of available studies highlights the need for further clinical research to translate this hypothesis into effective treatments and improve patient outcomes.

## Author contributions

BL: Writing – review & editing. DL: Writing – review & editing. AG: Writing – review & editing. PM-B: Writing – review & editing.
